# Catalytic Activity of Rare Earth Elements (REEs) in Advanced Oxidation Processes of Wastewater Pollutants: A Review

**DOI:** 10.3390/molecules28176185

**Published:** 2023-08-22

**Authors:** Lorenzo Saviano, Antonios Apostolos Brouziotis, Edith Guadalupe Padilla Suarez, Antonietta Siciliano, Marisa Spampinato, Marco Guida, Marco Trifuoggi, Donatella Del Bianco, Maurizio Carotenuto, Vincenzo Romano Spica, Giusy Lofrano, Giovanni Libralato

**Affiliations:** 1 Department of Biology, University of Naples Federico II, 80126 Naples, Italy; lorenzo.saviano@unina.it (L.S.); antonis.brouziotis@outlook.it (A.A.B.); edithguadalupe.padillasuarez@unina.it (E.G.P.S.); marisa.spampinato@unina.it (M.S.); marguida@unina.it (M.G.); donatelladelbianco09@gmail.com (D.D.B.); giovanni.libralato@unina.it (G.L.); 2 Department of Chemical Sciences, University of Naples Federico II, 80126 Naples, Italy; marco.trifuoggi@unina.it; 3 NBFC, National Biodiversity Future Center, 90133 Palermo, Italy; 4 CeSMA Advanced Metrological and Technological Service Center, University of Naples Federico II, 80126 Naples, Italy; 5 Department of Chemistry and Biology “Adolfo Zambelli”, University of Salerno, 84084 Fisciano, Italy; mcarotenuto@unisa.it; 6 Department of Movement, Human and Health Sciences, University of Rome “Foro Italico”, 00135 Rome, Italy; vincenzo.romanospica@uniroma4.it (V.R.S.); glofrano@unisa.it (G.L.)

**Keywords:** rare earth elements, catalytic activity, advanced oxidation processes, aqueous solutions

## Abstract

In recent years, sewage treatment plants did not effectively remove emerging water pollutants, leaving potential threats to human health and the environment. Advanced oxidation processes (AOPs) have emerged as a promising technology for the treatment of contaminated wastewater, and the addition of catalysts such as heavy metals has been shown to enhance their effectiveness. This review focuses on the use of rare earth elements (REEs) as catalysts in the AOP process for the degradation of organic pollutants. Cerium and La are the most studied REEs, and their mechanism of action is based on the oxygen vacancies and REE ion concentration in the catalysts. Metal oxide surfaces improve the decomposition of hydrogen peroxide to form hydroxide species, which degrade the organics. The review discusses the targets of AOPs, including pharmaceuticals, dyes, and other molecules such as alkaloids, herbicides, and phenols. The current state-of-the-art advances of REEs-based AOPs, including Fenton-like oxidation and photocatalytic oxidation, are also discussed, with an emphasis on their catalytic performance and mechanism. Additionally, factors affecting water chemistry, such as pH, temperature, dissolved oxygen, inorganic species, and natural organic matter, are analyzed. REEs have great potential for enhancing the removal of dangerous organics from aqueous solutions, and further research is needed to explore the photoFenton-like activity of REEs and their ideal implementation for wastewater treatment.

## 1. Introduction

Over the past few decades, conventional wastewater treatment plants (WWTPs) showed low efficiencies in removing contaminants of emerging concern (CECs) [1,2,3]. CECs encompass a wide range of substances, including pharmaceuticals, personal care products, and dyes [1,4].

The chronic release of these substances in water bodies represents a serious threat to human health and ecosystems and requires urgent advancements in wastewater treatment technologies [5,6,7] focusing also on the perspective of treated wastewater reuse.

Advanced oxidation processes (AOPs) evidenced potential revolutionary solutions in the development of integrated systems for the treatment of contaminated wastewater [1,8]. The mechanism of action of AOPs relies on the generation of reactive oxygen species (ROS) with strong oxidation capabilities, such as hydroxyl (OH^⦁^), sulfate (SO_4_^2−⦁^), and superoxide ion radicals (O_2_^−⦁^), which quickly degrade a wide range of organic compounds in CO_2_, H_2_O, and inorganic acids [9,10,11]. The addition of catalysts, such as heavy metals, can promote the development of such radicals and improve the process efficiency [12,13,14].

AOPs, particularly Fenton oxidation, are commonly carried out under acidic conditions, leading to the consumption of significant amounts of acid and base reagents [15,16]. The effectiveness of AOPs relies on the proper dosage of reactive hydroxyl (HO) radicals, ensuring the desired level of treatment is achieved [17,18]. A major limitation of AOPs is their relatively high cost, primarily due to the use of expensive chemicals and increased energy consumption [17]. Additionally, AOPs can result in the formation of unknown recalcitrant by-products, which may be more nephrotoxic than the original compounds, posing unresolved challenges [1,7]. While AOPs are effective in scavenging reactive radicals from non-target substances, they may not be suitable for certain categories of toxic compounds that resist degradation by HO radicals.

Recently, the application of rare earth elements (REEs) in the advanced oxidation process (AOP) has gained growing interest [19]. The REEs are a group of seventeen chemical elements in the periodic table, the 15 lanthanides from lanthanum (La) to lutetium (Lu), to which scandium (Sc) and yttrium (Y) are added since the latter tend to occur in the same mineral deposits with lanthanides and exhibit similar chemical properties. REE ions have a particular electronic structure compared to transition metal ions and are gaining great importance as Lewis acids in Green Chemistry because they can be used in aqueous solvents or water without any deactivation; they can be easily recovered from the reaction medium by extraction and can be used several times without loss of activity [19,20]. The redox properties of REEs depend on the arrangement of the surfaces and the shape and size of their crystals, and, for this reason, they can be improved by checking the nanostructure [21].

REEs exhibit unique physicochemical properties, such as high surface area, reactivity, and redox potential [22]. These properties make REEs suitable to be used in AOPs, where they can enhance the performance of the processes [23,24]. REEs can be used as catalysts to promote the generation of reactive oxygen species (ROS) when exposed to UV radiation in photocatalysis processes [25], to catalyze Fenton-like processes [26], and to enhance the efficiency of ozonation by increasing the production of hydroxyl radicals [27].

To date, most applications are still at lab scale and an increasing number of studies are being performed to fill the existing gaps in the usage of REEs for polluted water treatment. The main limitations to full-scale applications deal with high energy and capital cost.

This review focuses on the use of REEs as catalysts for the degradation of CECs from wastewater, since their use, which is currently in place for wastewater treatment, lacks processing and economic sustainability.

## 2. Methodology of Data Collection

The REE-assisted catalytic removal of CECs has substantially grown in recent decades, as retrieved in a Scopus search (keywords: rare earth elements, catalyst, and wastewater) updated on 20 December 2022.

**Figure 1:**
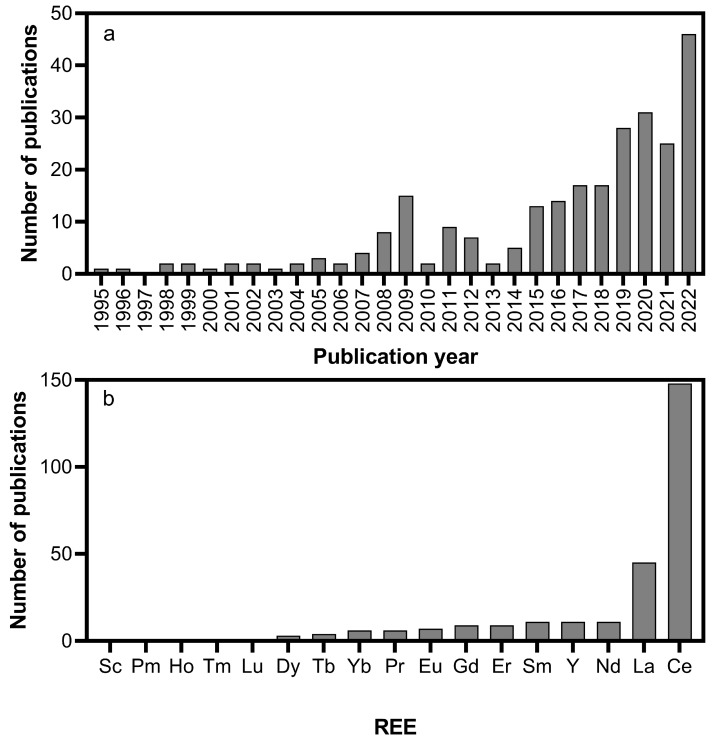
(**a**) The number of papers issued each year on catalytic removal of contaminants in wastewater treatment using REEs over the period of 1995–2022; (**b**) a cumulative number of publications for each REE over the period of 1995–2022.

We have collected 60 papers dealing with the degradation of different CECs from wastewater using REEs in AOPs. As shown in Figure 1a where the number of papers issued each year on catalytic removal of contaminants in wastewater treatment using REEs have been reported, the first publication was made in 1995 [28], which means that the field is quite new. Since 2015, the number of publications has been increasing each year. Figure 1b shows the cumulative number of publications for each REE over the period of 1995–2022. Most processes were carried out by using Ce and La. Cerium has received by far most of the attention (148 publications), followed by La (44), Y (10), and Nd (10). Five REEs (Sc, Ho, Tm, Lu, and Pm) and their possible effective role in this field have never been explored.

**Figure 2:**
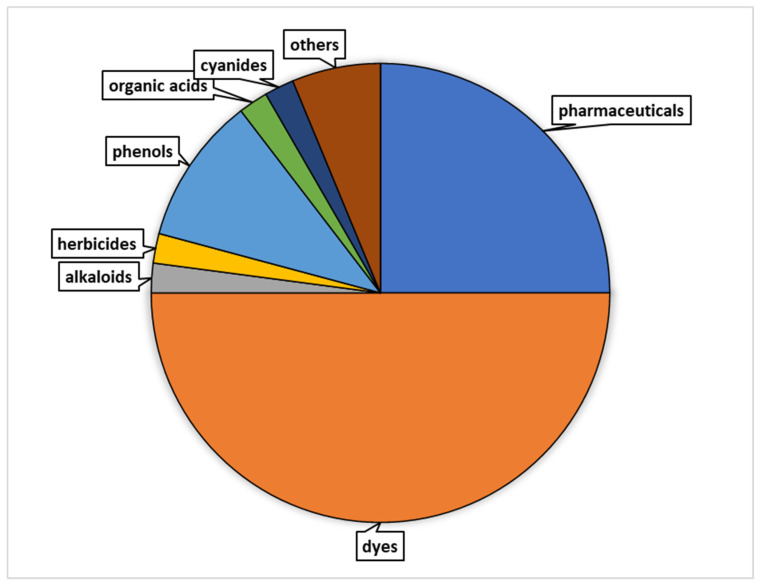
Percentage distribution of research papers on the utilization of REE catalysts for the degradation of different contaminant types.

Figure 2 presents the distribution of research papers that focused on the utilization of REE catalysts for the degradation of various types of contaminants. According to the data, pharmaceutical compounds and dyes accounted for the highest percentage of research papers, with approximately 33% and 42%, respectively. Alkaloids, herbicides, phenols, organic acids, cyanides, and other contaminants were also investigated to a lesser extent, each representing 3–8% of the papers. This distribution highlighted the varying levels of research emphasis on different categories of contaminants in relation to REE catalysts in the past.

The chemical, metallurgical, and physical properties of the REEs are governed by their electron configuration. In contrast to element Y, the lanthanides are characterized by the progressive filling of the 4f orbital [29]. Due to the low energy of these electrons, they do not directly participate in the bonding when forming compounds with other elements. As a consequence, this leads to a significant similarity in the chemical reactivity of the lanthanides. This uniform reactivity pattern is evident consistently throughout geochemical processes, rather than being unique to each individual element [29,30].

In Table 1, the essential details encompassing the atomic numbers and intricate valence shell electronic configurations pertaining to the distinctive set of REEs are provided.

## 3. AOP-Based REEs for CECs Removal

### 3.1. Pharmaceutical Degradation

Pharmaceutical toxic compounds can end up in wastewater via many routes, such as via their consumption by humans or their use in agriculture. Therefore, it is essential to discover effective ways for decomposing these hazardous compounds in wastewater. The use of REEs has been shown by several studies to be potentially useful. Numerous researchers utilized REEs in various forms as heterogeneous catalysts in AOPs (Table 1).

Nie et al. [31] carried out a Fenton-like reaction, at 30 °C, to take complete degradation of the antibiotic sulfamethoxazole (SMX), in the simultaneous presence of LaFeO_3_ and H_2_O_2_ at pH values ranging between 5.5 and 7.14. It was found that the SMX removal efficiency increased with the increase of the catalyst load and then reached a constant value when the LaFeO_3_ concentration was above 1.4 g/L. Furthermore, it was investigated that the LaFeO_3_ could be reused for at least 10 cycles and the reused catalyst kept the catalytic activity nearly as efficient as the fresh one. Ref. [32] investigated the photocatalytic activity for degradation of carbamazepine and caffeine using MIL-125-NH_2_, LaFeO_3_, and LaFeO_3_/MIL-125-NH_2_ photocatalysts under solar simulator at ambient temperatures. LaFeO_3_/MIL-125-NH_2_ composite exhibited higher carbamazepine and caffeine degradation than MIL-125-NH_2_ MOF and LaFeO_3_ perovskite and this is highlighted through the calculation of pseudo-first-order kinetics equation. LaFeO_3_/MIL-125-NH_2_ composite photocatalysts prepared by the self-assembly method have shown excellent and promising results in the degradation of pharmaceutical compounds.

Chen et al. (2022) [33] used LaFeO_3_/lignin-biochar (LFO/LG) catalysts prepared by a sol–gel pyrolysis to evaluate the degradation of ofloxacin (OFX) under visible light irradiation. The pure LaFeO3 (LFO) and the pure lignin-biochar (LG) were tested and prepared under the same conditions to compare which one had the best performance. The whole process was operated at a constant temperature in the presence of H_2_O_2_ (30 wt%) and all experiments were carried out under different initial pH, revealing later an optimal photodegradation efficiency at pH values above 6. In addition, the morphology, microstructure, energy band structure, and photoelectrochemical behavior of the composite catalysts were characterized and the relationship with catalytic performance was explained. Pure LFO has almost no adsorption of OFX when the system was left in the dark to reach a saturation adsorption state and the degradation efficiency for LFO is only 53.4% after a 75-min degradation reaction. Instead, LFO-LG samples showed a significant adsorption effect on OFX, and the degradation efficiency was also improved up to 95.6%.

In the study of [34], the electrochemical degradation of dipyrone using cerium catalysts was not effective during a two-hour electrolysis. However, the use of a CeO_2_/C gas diffusion electrode degraded all of the dipyrone in 20 min with 26% mineralization at −1.3 V, and after only 5 min with 57% mineralization at −1.1 V when the Fenton process was employed. The authors declared that ceria acts as an oxygen buffer leading to an increase in the local oxygen concentration, facilitating H_2_O_2_ formation and consequently improving the dipyrone degradation. FeCeO_x_ can remove 83% of diclofenac in a heterogenous Fenton process and can be reused due to its chemical stability [35].

A heterogeneous Fenton catalyst, Ce^0^-Fe^0^-reduced graphene oxide (Ce–Fe–RGO), showed good catalytic performance and adsorption of sulfamethazine [13]. Ferrum-doped CeO_2_ nanosheets with various Fe/Ce ratios were evaluated for the Fenton-like degradation of salicylic acid (SA). The 2% Fe-doped CeO_2_ nanosheets showed the highest concentration of surface oxygen vacancies and catalytic activity under the optimum reaction conditions due to the complex adsorption of SA and H_2_O_2_ on the surface Ce^3+^. The catalytic activity of the nanosheets could be recovered, and so they could be reused in new cycles [36]. A surface of graphite felt loaded with CeO_x_ accelerated the mineralization of carbamazepine via an E-peroxone process [37].

The Fe^0^/CeO_2_ nanocomposite enhanced the removal of tetracycline (TC) in the Fenton oxidation process and could be reused for further cycles. The authors suggested a synergistic effect between nanoscale Fe^0^ and CeO_2_ [19].

Hu et al. [38] tried out for the first time the use of cobalt (Co) and REE gadolinium (Gd) to investigate the removal of two antibiotics from wastewater, ciprofloxacin (CIP) and tetracycline. In particular, the sponge-like structure of Co- and Gd-modified biochar (MBC) was conducive to studying its adsorption capacity due to the presence of metal oxides and functional groups. Furthermore, the influence of pH on the absorption of antibiotics was studied by varying the pH between 3 and 11. It was observed that at pH 9, the antibiotic adsorption capacity of MBC was greatly increased. These data suggested that MBC is an innovative and effective adsorbent for the removal of antibiotics from complex contaminated water.

Luan et al. [39] synthesized photocatalysts Er_2_FeSbO_7_, BiTiSbO_6_, or N-TO by solid-state method to degrade enrofloxacin (ENR), a common antibiotic able to effectively kill Gram-positive and Gram-negative bacteria and mycoplasma under visible light irradiation. A new photocatalyst provided by Er_2_FeSbO_7_/BiTiSbO_6_ heterojunction (EBH) was prepared for the first time by the thermal solvent method and tested. It was observed that the photocatalytic activity among the four photocatalysts was as follows: EBHP > Er_2_FeSbO_7_ > BiTiSbO_6_ > N-TO. Therefore, it can be concluded that the EBHP process can be a potent method for treating pharmaceutical wastewater that is polluted by ENR.

In general, the studies highlighted the use of diverse catalysts to improve the efficiency of removing pharmaceutical compounds. Each study focused on targeting specific pharmaceutical compounds; this targeted approach allowed for a more accurate evaluation of the catalyst’s performance against particular contaminants. Among the studies conducted on CIP removal, the highest removal percentage (>99%) within a timeframe of 180 min was achieved by the study utilizing the MBC catalyst [38].

The pH can play a significant role in the reactivity and adsorption behavior of both the catalyst and the target compound. The studies reported a range of pH values, including pH 4, 5, 6.48, and 9, indicating that different pH levels can be effective depending on the specific catalyst and target compound.

Furthermore, the temperature of the reaction can influence the reaction kinetics and the stability of the catalyst. It was generally observed that moderate temperatures (20–25 °C) were preferred to strike a balance between reaction rates and energy consumption.

The pharmaceutical degradation studies highlighted the potential of Fenton-based processes for wastewater treatment, showcasing the reusability of FeCeO_x_ [33], the catalytic performance and adsorption properties of Ce-Fe-RGO [13], and the degradation capabilities of ferrum-doped CeO_2_ nanosheets [19]. The results emphasize the versatility and effectiveness of Fenton processes in removing pharmaceutical compounds, offering promising solutions for the treatment of wastewater contaminated with these contaminants.

### 3.2. Dye Degradation

Wastewater from the textile industry contains residual dyes which are hardly biodegradable. Advanced Oxidation Processes (AOPs) are alternative techniques for the destruction of dyes and many other organic products in wastewater and effluents. In Table 2, the degradation efficiency of various dyes using REE catalysts is highlighted, including the type of contaminant, initial concentration, reaction conditions, and the corresponding degradation percentage achieved by the REE catalysts.

The presence of La in the La–Fe montmorillonite (La–Fe MMT) composite enhanced the degradation efficiency of the organic dyes Rhodamine B (RhB) and methylene blue (MB) and resulted in a little iron leaching, consequently increasing the lifetime of the system. Due to its low cost, efficient reactivity, high stability, and low metal leaching, it seems a great potential as a green catalyst for the heterogeneous Fenton-like degradation of hazardous dyes [40]. FeOCl doped with Y or La showed efficient Fenton catalytic activity for ibuprofen degradation under simulated solar light [41]. Tungsten oxide composites (WO_3_) doped with La, Gd, or Er showed an enhanced photocatalytic degradation of organic dyes compared to the pure composites [42].

Huang et al. (2014) [43] doped hydroxyl FeAl intercalated montmorillonite with La or Ce and saw an improvement in the photo-Fenton catalytic oxidation of Reactive Blue 19 under natural sunlight irradiation. The photo-Fenton activity of pristine CoFe_2_O_4_ on the oxidation of five organic dyes was enhanced by inserting a very small quantity of La or Ce cations into its spinel structure in the presence of two different oxidants [44].

Divya and Renuka (2015) [45] doped nanoceria with five different elements (Cu, Fe, Zr, Dy, and La) and investigated the heterogenous Fenton-like oxidation of MB. CuO showed the highest catalytic activity, which is attributed to the synergistic effect between copper and ceria. The Cu–Mn/CeO_2_/SBA-15 catalysts can efficiently degrade (>99%) high-concentration dye pollutants. The excellent catalytic performance could be attributed to the well-dispersed catalytic nanoparticles inside the mesopores and the catalytic synergic effect of CeO_2_ [46]. Xie, Yibing, and Chunwei Yuan [47] showed that Ce^4+^-TiO_2_ sol and nanocrystallites can photodegrade the reactive brilliant red dye (X-3B) with the dye/Ce^4+^-TiO_2_/visible-light reaction system.

Singh et al. (2016) [48] prepared several Cu/zeolite Y catalysts to test the decolorization, degradation, and mineralization of recalcitrant diazo dye (Congo red) in a heterogeneous Fenton-like process. The catalyst with 7.5 wt% Cu showed the maximum degradation, decolorization, and mineralization of 93.58%, 95.34%, and 79.52% after 2.5, 2, and 4 h, respectively, along with reproducible activity for three cycles. The addition of Y on the surface of BiW_2_O_6_ composites enhanced the degradation of RhB compared to the individual composites under visible light illumination, by producing holes (h^+^) and superoxide radicals [49].

Seroglazova et al. (2022) [50] showed that Praseodymium orthoferrite (PrFeO_3_) nanopowders can achieve 100% efficiency of photo-Fenton-like degradation of methyl violet. The PrFeO_3_/CeO_2_-based nanocomposites can degrade methyl violet (MV) under the presence of visible light via a photocatalytic Fenton-like activity. These nanoparticles can be reused due to their stability and be separated from the reaction solution [51]. In the study of [52], Pr-CdWO_4_ nanoparticles could degrade the toxic synthetic azo dye Remazol Black B by 93.9% at optimal conditions.

Nd-doped ZnO nanoneedles can degrade MB under UV light. Especially 1% Nd can degrade 2.5 times more with respect to undoped ZnO [53]. The LaFeO_3_ perovskites exhibit better photocatalytic activity towards the oxidative degradation of dye molecules when doped with Nd, Eu, Gd, or Dy [54].

Keerthana et al. (2021) [55] showed that zinc ferrite (ZnFe_2_O_4_) nanoparticles doped with 2% Samarium (Sm) can remove 65% of MB dye with the help of visible light irradiation within 1 h and, due to their stability, could be reused for more than three cycles. In the study of [56], Sm-doped CeO_2_ nanoparticles demonstrated a very efficient photodegradation of UV-irradiated rose bengal dye. Wang et al. (2017) [57] showed that Sm-doped Bi_2_MoO_6_ photocatalysts could degrade more effectively RhB with respect to the individual particles.

The europium-nitrogen (EueN) co-doped TiO_2_/Sepiolite nanocomposites, prepared through microwave-hydrothermal treatment, can be used to effectively eliminate dyestuff in real wastewater treatment. In particular, in order to evaluate the degradation of an anionic dye, Orange G, the photocatalytic activities were conducted under solar irradiation. The Eu-N co-doped TiO_2_/Sep NCs, with various amounts of Eu^III^, photocatalysts exhibited improved photoactivity as compared to mono-doped and undoped samples [58]. In other studies, europium, in the +2 and +3 oxidation state, has been used as a dopant in VOC degradation over metal oxide catalysts such as TiO_2_ for photocatalytic oxidation of RhB and MB [59,60] and BiVO_4_ for photocatalytic oxidation of methyl orange [61]. EuFeO_3_ nanoparticles have photo-Fenton-like catalytic activity, as indicated by the efficient decolorization of RhB aqueous solution under visible-light irradiation, and they can be recovered and reused due to their excellent photocatalytic stability [62]. Xu et al. (2014) [63] showed that the addition of Eu^3+^ ions on the surface of Bi_2_WO_6_ composites enhances the photocatalytic degradation of RhB under visible light irradiation by producing hydroxyl radicals.

Sharmin et al. (2022) [64] synthesized 10% Gd-doped BiFeO_3_ nanoparticles (BGFO) via a simple and cost-effective hydrothermal technique at a lower reaction temperature of 160 °C. Following this approach, the synthesized nanoparticles exhibited photocatalytic activity towards the degradation of hazardous industrial pollutants, such as RhB and MB pharmaceutical pollutants such as ciprofloxacin (CIP) and levofloxacin (LFX) under simulated solar irradiation. The photocatalyst BGFO demonstrated 96% and 97% degradation of RhB and MB within 240 and 180 min of solar irradiation, respectively, and about 80% and 79% degradation of CIP and LFX were obtained within 240 min in the same conditions. Similarly, through the hydrothermal method, Sn-Gd_2_O_3_ nanomaterial was prepared and mixed with a hot aqueous solution (T > 60 °C) of gelatin polymer, followed by cross-linking, for the efficient reduction of water pollutants [65]. The catalytic activity of Sn-Gd_2_O_3_ NPs was analyzed against different dyes, such as 4-nitrophenol (4-NP), 2,6-dinitrophenol (2,6-DNP), 2-nitrophenol (2-NP), MB, methyl orange (MO) and congo red, the presence of a strong reducing agent, NaBH_4_. These latter two anionic azo dyes are used as indicators as well as in the food, textile, paper and pulp, cotton, silk, plastic, cosmetic, pharmaceutical, rubber, printing, and wool industries [66]. Therefore, this scientific approach has led to the fabrication of a new hydrogel nanocomposite for the removal of water pollutants, especially in the reduction of the activity of industrial dyes. Composites of Bi_2_MoO_6_ showed an enhanced photodegradation of RhB and TC when doped with Gd and Pt ions, by producing hydroxyl radicals, holes in the valence band (h^+^), and superoxide radicals [67]. Yu et al. (2016) showed that Bi_2_MoO_6_ nanoplate crystals perform a better photocatalytic degradation of RhB when doped with Gd^3+^ ions, with the production of hydroxyl radicals as a proposed mechanism of action [68]. Wu et al. (2019) [69] showed that the TiO_2_ NPs demonstrated an enhanced photocatalytic degradation of MB and RhB when doped with Gd^3+^ ion or Gd_2_O_3_.

Vinosha et al. (2022) [70] showed that zinc ferrite (ZnFe_2_O_4_) nanocrystals doped with dysprosium (Dy) exhibit a photo-Fenton activity against MB with an efficiency of 97.3% within 45 min. This ferrite can act as a magnetic recyclable catalyst even after five cycles with an insignificant lessening of elements.

Liang et al. (2006) [71] showed that Er^3+^-TiO_2_ catalysts had higher adsorption equilibrium constants and better adsorption capacity than pure TiO_2_, and the degradation and mineralization of orange I under both UV radiation and visible light were more efficient with Er^3+^-TiO_2_ catalyst than with pure TiO_2_.

Tikhanova et al. (2021) [72] showed that the heterojunction h-YbFeO_3_/o-YbFeO_3_ photocatalyst demonstrates an enhanced photo-Fenton decolorization of MV under visible light compared to pure o-YbFeO_3_. A novel developed 2.0%Yb/2.0%Er/2.0%Pr-BiW_2_O_6_ catalyst could degrade the Congo red dye, TC, RhB, and MB by 90.2%, 52.3%, 95.5%, and 64.6%, respectively, under visible light illumination. The proposed mechanism of action was the promoted reactive oxide species production and photocarrier separation [73].

The addition of Ln_2_O_3_ (Ln = Sm, Eu or Tb) on the surface of BiVO_4_ composites enhanced the degradation of RhB with respect to the individual composites, by producing superoxide radicals, holes (h^+^), and hydroxyl radicals [74]. In the study of [75], the Bi2WO6 photocatalyst showed enhanced performance in the degradation of RhD when doped with the combination of Tb/Eu, Dy/Sm, or Er/Nd. The proposed mechanism of action was the production of hydroxyl radicals, holes (h^+^), and superoxide radicals.

Among the catalysts discussed, the most promising results for the removal of dyes were obtained with nanoceria doped with Cu, Fe, Zr, Dy, or La, which achieved a remarkable removal efficiency of over 99% MB [45]. These catalysts demonstrated a high level of effectiveness in degrading and eliminating MB from wastewater, showcasing their potential for applications in water treatment. However, it is important to consider that the performance of catalysts can be influenced by various factors, such as reaction conditions and the specific dye compound being targeted. Optimal results may vary depending on these factors and further exploration and optimization are necessary to discover even more efficient catalysts for MB removal. By improving catalyst design, fine-tuning reaction conditions, and exploring novel materials, it may be possible to achieve even higher removal efficiencies and enhance the overall sustainability and efficiency of water treatment processes.

### 3.3. Degradation of Other Target Pollutants

With the rapid development of industrialization, wastewater has increasingly enriched itself with various substances which often include, in addition to those already extensively discussed above, alkaloids, herbicides, phenols, etc. Unconventional wastewater treatment methods are presented below based on photocatalysis and Fenton-like reaction using REEs for the abatement of these molecules. In the first-ever study using REEs for removing pollutants from aqueous solutions, [33] studied the use of solid compounds of Y(III), La(III), Ce(III), Nd(III), Sm(III), Gd(III), and Ce(IV) in the form of oxides, hydrous oxides and basic carbonates for the removal of fluoride ions, and only Ce(IV) was capable of removing them effectively without a significant dissolution even at pH 2.

Fouad et al. (2021) [76] tried to modify the LaFeO_3_ catalyst with Ti (Ti-LaFeO_3_) by developing photocatalysis and photo-Fenton processes under UV and visible light for the degradation of carbofuran (CBF). It was observed that about 20% of CBF was degraded by H_2_O_2_ without catalyst loading under UV irradiation. In the presence of UV light and the absence of H_2_O_2_, the CBF degradation was 59%, which was mainly due to the photocatalysis process induced by the illumination of Ti-LaFeO_3_. The degradation of CBF was 73% under metal-halide lamp irradiation in the system of Ti-LaFeO_3_/H_2_O_2_, which confirms the activity of the catalyst under visible light. Moreover, the degradation was improved to 89.5% when this lamp was replaced by UV light. Heterogeneous catalytic wet peroxide oxidation (CWPO) was proposed in this work, aiming to obtain degradation rates and faster mineralization of recalcitrant pollutants like CBF [77]. The results showed that the efficiency of the CWPO process by Ti-LaFeO_3_ is higher than other photocatalytic processes used in the literature for the degradation of CBF. Furthermore, the catalyst could maintain its catalytic activity for five consecutive cycles. In the study of [78], the La-doped TiO2 photocatalyst could degrade acetone and NO under visible light by 38% and 98%, respectively.

Gogoi et al. (2017) [79] used Fe_3_O_4_-CeO_2_ nanocomposite materials as a Fenton-like heterogeneous catalyst for the degradation of catechol. Fe_3_O_4_-CeO_2_ (15 wt%) showed the maximum degradation of catechol compared to the other prepared catalysts. The catalyst could also be recovered and used for five consecutive cycles with excellent catalytic activity. Cerium chloride exhibited radical production in the presence of hydrogen peroxide, as assessed by relaxation of supercoiled plasmid DNA and the production of radical cation of 2,2′-azinobis-(3-ethylbenzthiazoline-6-sulfonic acid), and so it is proposed that cerium is capable of redox-cycling with peroxide to generate damaging oxygen radicals [80]. Orge et al. (2012) [81] tested ceria and Ce-based mixed oxides (Ce-Sm, Ce-La, and Ce-Zr with different compositions) as catalysts in the ozonation of oxalic acid, aniline, and the textile dye C. I. Reactive Blue 5. CeO_2_-ZrO_2_ catalyst with higher than 25 wt.% of ZrO_2_ presented the best catalytic oxidation of oxalic acid, which may be related to the larger percentage of the Ce (III) species on their surfaces. On the other hand, the ozonation catalyzed by 25 wt.% of Zr, Sm, or La oxides with Ce oxide or by Ce oxide did not present significant differences. In addition, the association of O_3_ and all mentioned catalysts leads to an improved TOC removal from the dye and almost complete mineralization in all cases after about two hours. The highest mineralization rate of aniline was achieved by CeO_2_ and Ce_0.75_Zr_0.25_O_2_ with the latter being the sample with the largest amount of oxygen vacancies number in its structure. Cerium nanotubes catalysts (CeNTs) doped with different amounts of Nb_2_O_5_ (0–10 wt.%) were used for the catalytic reduction of NO_x_ with NH_3_ (NH_3_-SCR) in the presence of CH_2_Cl_2_. The 10 wt.% Nb-CeNTs catalyst presented the best oxidation of NH_3_-SCR and degradation efficiency of CH_2_Cl_2_ among the prepared catalysts, due to its abundant surface oxygen species and acid sites, superior redox capability, the interaction between Nb and Ce, higher ratio of Nb^4+^/(Nb^5++^ Nb^4+^) and Ce^3+^/(Ce^3++^ Ce^4+^), and the special tubular structure of the Ce nanotube [82].

A magnetic nanoscaled Fe_3_O_4_/CeO_2_ composite can degrade 4-chlorophenol and be reused for six successive cycles [83].

A ZnO nanoparticle doped with 2% Ce can catalyze the oxidation of cyanide and subsequently of cyanate as well under UV-A light or natural sunlight [84].

Sharma et al. (2016) [44] doped cobalt ferrite (CoFe_2_O_4_) with La or Ce and observed an enhancement in its photo-Fenton degradation properties of various organic pollutants using two different inorganic oxidants: hydrogen peroxide (H_2_O_2_) and potassium peroxymonosulphate (KHSO_5_), with respect to pure cobalt ferrite. The doped catalyst could also be reused in wastewater treatment without a decline in its catalytic activity due to its chemical stability.

Samarium-doped ZnO nanorods (Sm/ZNRs) can exhibit a higher photocatalytic degradation of phenol aqueous solution under visible light irradiation than that of the pure ZNRs due to their high charge separation efficiency and OH^•^ generation ability. These nanorods could also be reused for more cycles [85].

Cymes et al. (2020) [86] doped cryptomelane with europium via either co-precipitation or ion exchange in order to promote the catalytic oxidation of ethanol. Eu-doping by co-precipitation generally improves catalytically advantageous physical and chemical properties whereas Eu-doping by ion exchange shows deleterious effects.

Composites of Bi_2_MoO_6_ showed an enhanced photodegradation of 4-Chlorophenol when doped with Gd and Pt ions, by producing hydroxyl radicals, holes in the valence band (h^+^), and superoxide radicals [49]. In the study of [63], the Bi_2_WO_6_ photocatalyst showed enhanced performance in the degradation of phenol when doped with the combination of Tb/Eu, Dy/Sm, or Er/Nd. The proposed mechanism of action was the production of hydroxyl radicals, holes (h^+^), and superoxide radicals.

Table 2 shows which REEs have been studied for photocatalysis and which for Fenton-like catalysis.

Table 3, Table 4 and Table 5 below summarize all the catalyst-doped REEs divided and ordered according to publication date.

## 4. Potential Environmental Impacts of REE Catalysts

As for the use of REEs in AOPs, different perspectives are to be considered. Further improvement needs to be implemented in water treatments; as of now, several methods have been adopted that increase efficiency and provide better handling of waste, such as Fenton-like oxidation processes [87]. Studies focused on the use of lanthanides have demonstrated an improvement in wastewater treatments, as their use can produce on average 30% less sludge compared to other alternatives such as iron and aluminum [88]. The use of REEs could represent an alternative for reducing wastelands generated by sludge [88] and improving the removal of organic pollutants by enhancing photocatalytic activity [23].

Moreover, care should be taken to avoid the loss and transfer of particles into the environment, contributing to the anthropogenic input of REEs. The authors of [89] found that the mixture of lanthanides used in wastewater treatment had an antagonistic behavior, which could reduce their environmental impact. However, up to now, the knowledge of the toxicity of REEs is very limited, as the environmental fate is highly dependent on the system, such as the presence of organic matter, pH, and presence of cations [90] which influence the speciation, which, by extension influences the bioaccumulation and bioavailability. Because of the lack of knowledge regarding the potential effects of REEs, the regulation for the discharge of REEs is still lacking. In contrast, many restrictions regarding the presence of organic pollutants have been applied globally [91].

## 5. Conclusions

The study of REEs for their potential use in removing toxic organic compounds from aqueous solutions is a relatively recent field, with the first publication on this topic occurring only 28 years ago. Among the REEs, cerium has been extensively studied for its photo Fenton-like catalytic activity against organic pollutants, followed by lanthanum. However, the remaining REEs have received little attention, with five of them having never been studied in this context.

REEs have been shown to be promising for removing hazardous organic compounds from aqueous solutions, such as wastewater. The effectiveness of REEs in this regard depends mainly on the presence of oxygen vacancies and the concentration of REE ions in the catalysts. Specifically, the metal oxide surfaces promote the decomposition of hydrogen peroxide, which leads to the formation of hydroxide species responsible for the degradation of organic compounds.

Moreover, AOPs, which include REE-combined catalysts and other parameters, should be chosen based on the nature of the contaminants and the acid-base properties of the wastewater to be treated.

Due to their exceptional properties, REEs have the potential to revolutionize wastewater treatment. However, achieving the production of clean water without generating traces of persistent toxins remains a challenging goal.

## Figures and Tables

**Table 1:** Atomic Number and Valence Shell Electronic Configuration of the Rare Earth Elements.

Element	Atomic Number	Valence Shell Electronic Configuration
**Y**	39	[Ar] 3d1 4s2
**La**	57	[Ar] 4d1 5s3
**Ce**	58	[Xe] 4f1 5d1 6s2
**Pr**	59	[Xe] 4f3 5d0 6s2
**Nd**	60	[Xe] 4f4 5d0 6s2
**Pm**	61	[Xe] 4f5 5d0 6s2
**Sm**	62	[Xe] 4f6 5d0 6s2
**Eu**	63	[Xe] 4f7 5d0 6s2
**Gd**	64	[Xe] 4f7 5d1 6s2
**Tb**	65	[Xe] 4f9 5d0 6s2
**Dy**	66	[Xe] 4f10 5d0 6s2
**Ho**	67	[Xe] 4f11 5d0 6s2
**Er**	68	[Xe] 4f12 5d0 6s2
**Tm**	69	[Xe] 4f13 5d0 6s2
**Yb**	70	[Xe] 4f14 5d0 6s2
**Lu**	71	[Xe] 4f14 5d1 6s2

**Table 2:** REEs studied for simple photocatalysis and Fenton-like photocatalysis.

Type of Reaction	REEs Studied
Photocatalysis	Y, La, Ce, Nd, Sm, Eu, Gd, Tb, Dy, Er, Yb
Fenton-like catalysis	Y, La, Ce, Pr, Nd, Eu, Gd, Dy, Yb

**Table 3:** REE catalysts used for pharmaceutical degradation.

Catalyst Doped REE	Target	Dose of Catalyst	Dose of Target	Time (min)	Conditions	Proposed Mechanism	Removal (%)	Reference
LaFeO_3_	Sulfamethoxazole	1.4 g/L	3 mg/L	120	pH = 6.48, T = 30 °C	Production of •OH and superoxide radicals (O_2_^−^/HOO)	100%	[31]
LaFeO_3_/MIL-125-NH_2_	Carbamazepine (CBZ) and Caffeine (CAF)	250 mg/L	5 mg/L CBZ and 1 mg/L CAF	60	Visible light	Production of •OH and O_2_^−^	74% CBZ 87% CAF	[32]
LaFeO_3_/lignin-biochar (LFO/LG)	Ofloxacin (OFX)	250 mg/L	30 mg/L	75	Visible light, addition of H_2_O_2_	Production of •OH	95.60%	[33]
CeO_2_/C gas diffusion electrode (GDE)	Dipyrone	-	100 mg/L	20	-	Production of •OH	100%	[34]
FeCeO_x_	Diclofenac	0.5 g/L	20 mg/L	40	pH = 5, addition of H_2_O_2_, ambient temperature	Production of •OH	83%	[35]
CeO_2_ nanosheets doped with Fe	Salicylic acid	250 mg/L	50 mg/L	120	Addition of H_2_O_2_, pH = 4, T = 55 °C	Production of superoxide radicals (O_2_^−^/HOO)	96% by 2wt% Fe-CeO_2_	[36]
CeO_x_ modified graphite felt	Carbamazepine	-	10 mg/L	60	pH = 5, T = 25 °C	Production of •OH	69.40%	[37]
Sponge-like structure of Co- and Gd-modified biochar (MBC)	Ciprofloxacin (CIP)/Tetracycline (TC)	1.1 g/L	20 mg/L	180	pH = 9	-	99% of CIP or TC	[38]
Er_2_FeSbO_7_/BiTiSbO_6_ heterojunction (EBH) catalyst	Enrofloxacin (ENR)	0.75 g/L	0.025 mM	150	Visible light, T = 20 °C,	Production of •OH	99,16%	[39]
FeOCl doped with Y or LA	Ibuprofen	0.5 g/L	5 mg/L	20	Neutral pH, addition of H_2_O_2_, room temperature, dark	Production of •OH	84–82% by 0.9wt% FeOCl/La and 1.2wt% FeOCl/Y, respectively	[40]
Gd doped BiFeO_3_ nanoparticles (BGFO)	Ciprofloxacin (CIP)/Levofloxacin (LFX)	-	-	240	Solar illumination	Production of O_2_^−^	80%/79%	[64]

**Table 4:** REE catalysts used for dyes degradation.

Catalyst Doped REE	Target	Dose of Catalyst	Dose of Target	Time (min)	Conditions	Proposed Mechanism	Removal (%)	Reference
La–Fe MMT	Rhodamine B (RhB)/Methylene Blue (MB)	1 g/L	100 mg/L	60	Neutral pH, addition of H_2_O_2_	Production of •OH	97% MB 96% RhB	[40]
Tungsten oxide composites (WO_3_) doped with La, Gd, or Er	Organic dyes	300 mg/L	-	90	Visible light,	Production of O_2_^−^	98% for MB and >90% for MT, MO, TC and CV by 2% Gd-doped WO_3_	[42]
FeAl/Ce-Mts and FeAl/La-Mts	Reactive Blue 19	0.5 g/L	0.12 mM	180	Sunlight, addition of H_2_O_2_, pH = 3.0	Production of •OH	100% by FeAl/Ce1.0-Mt and 99.7% with FeAl/La1.0-Mt	[43]
CoFe_2_O_4_ doped with La or Ce	Remazol black 5, remazol brilliant yellow, *o*-nitrophenol, *m*-nitrophenol (MNP) and *p*-nitrophenol	0.5 g/L	-	ott-30	Visible light, room temperature, pH = 2.5, addition of H_2_O_2_	Production of •OH	-	[44]
Nanoceria doped with Cu, Fe, Zr, Dy or La	MB	80 mg/L	55.2 mg/L	420	pH = 9.6, T = 27 °C, UV light	Production of •OH	>99% by CuO/CeO_2_	[45]
Cu-Mn/CeO_2_/SBA-15	RhB	200 mg/L	2 g/L	210	pH = 7.0, addition of H_2_O_2_, atmospheric pressure and constant temperature	Production of •OH	0.99	[46]
Ce_4_^+^-TiO_2_ sol and nanocrystallites	Brilliant red dye (X-3B)	1 g/L	100 mg/L	120	Visible light	Production of •OH and O_2_^−^	83.1% by Ce_4_^+^-TiO_2_ nanocrystallines and 99.9% by Ce_4_^+^-TiO_2_ sol	[47]
Cu/zeolite Y	Congo red (CR)	1 g/L	0.143 mM	150	Addition of H_2_O_2_, pH = 7.0	Production of •OH	93.58% by 7.5wt% Cu	[48]
PrFeO_3_	Methyl violet (MV)	250 mg/L	23.2 mg/L	60	Addition of H_2_O_2_, visible light	Production of •OH	-	[50]
PrFeO_3_/CeO_2_-based nanocomposites	MV	250 mg/L	23.2 mg/L	30	Visible light, addition of H_2_O_2_	Production of •OH	80.1% by 9 wt% CeO_2_	[51]
Pr-CdWO_4_ NPs	Remazol Black B	350 mg/L	100 mg/L	100	pH = 3.0, T = 25 °C, addition of enhancers	Production of •OH	93.90%	[52]
ZnO nanoneedles doped with Nd	MB	5 g/L	10^−5^ M	300	UV light	Production of O_2_^−^	92% by 1% Nd-doped ZnO	[53]
LaFeO_3_ perovskites doped with Eu, Gd, Dy, or Nd	Safranine-O and remazol brilliant yellow	0.5 g/L	15 mg/L SO and 60 mg/L RBY	20, 35, 35, 20	Visible light, pH = 2.0, adding H_2_O_2_	Production of •OH	More than 90%	[54]
ZnFe_2_O_4_ nanocrystals doped with Sm	MB	0.1 g/L	1 mg/L	60	Visible light	Production of •OH	65% by 2 wt% Sm	[55]
Sm doped CeO_2_ nanoparticles	Rose bengal dye	1 g/L	5 mg/L	90	UV light	Production of •OH	84% by 4 wt% Sm and 89% by 6 wt% Sm	[56]
Eu-N co-doped TiO_2_/Sepiolite NCs	Orange G	0.8 g/L	10 mg/L	540	Visible light, pH = 3.0	Production of •OH	More than 98% by 0.6 wt% Eu	[58]
Eu^3+^–TiO_2_	RhB	2 g/L	10^−5^ M	30	UV light	Formation of a complex between the doped lanthanide ions and substrates	96%	[59]
Eu^2+^–TiO_3_	MB	0.125 M	2 × 10^−5^ M	60	UV light, pH = 8.3	Induced surface plasmon resonances	-	[60]
Eu^2+^,^3+−^BiVO_4_	Methyl orange (MO)	2 g/L	10 mg/L	180	Visible light	Trapping of photogenerated electrons in the catalyst by Eu dopant	93.6% by 1.46 wt% Eu	[61]
EuFeO_3_ NPs	RhB	1 g/L	5 mg/L	180	Visible light, room temperature, addition of H_2_O_2_	Production of •OH	71%	[62]
Gd doped BiFeO_3_ NPs (BGFO)	RhB/MB	-		240/180	Solar illumination	Production of O_2_^−^	96%/97%	[64]
Sn-Gd_2_O_3_ NPs	4-NP, 2,6-DNP, 2-NP, MB, MO and CR	8 g/L	0.07 mM	18	Addition of NaBH_4_	Electron transfer from the catalyst to the dye	-	[65]
ZnFe_2_O_4_ nanocrystals doped with Dy	MB	1 g/L	20 mg/L	45	Visible light, addition of H_2_O_2_	Production of •OH	97.30%	[66]
Er^3+^–TiO_2_	Orange I	1 g/L	6 × 10^−5^ M	60	T = 25 °C, UV light or visible light, pH = 7.0	Production of •OH	About 100% under UV and about 80% under visible light by 1.5 wt% Er	[70]
Heterojunction h-YbFeO_3_/o-YbFeO_3_	MV	125 mg/L	0.25 mM	180	Visible light, addition of H_2_O_2_	Production of •OH	64%	[72]
Yb/Er/Pr-Bi2WO6 catalyst	CR/TC/RhB/MB	1 g/L	-	60	Visible light	Reactive oxide species production and photocarrier separation	90.2%/52.3%/95.5%/64.6%	[73]
BiVO4 composites doped with Ln2O3 (Ln = Sm, Eu, or Tb)	RhB	1 g/L	10 mmol/L	210	Visible light, 25 °C	Production of •O_2_^−^, holes in the valence band (h^+^), and •OH	55.1%, 51.8%, and 57.3% by 2% Sm2O3/BiVO4, 2% Eu2O3/BiVO4, and 10% Tb2O3/BiVO4, respetively	[74]
Y-Bi2WO6 photocatalyst	RhB	1 g/L	0.01 mM	240	Visible light	Production of holes (h^+^) and O_2_^−^	85% by 1% Y-Bi2WO6	[49]
Gd/Pt-Bi_2_MoO_6_ composites	RhB/TCs	1 g/L	12 mg/L RhB/20 mg/L TCs	80 for RhB and 90 for TCs	UV visible light	Production of •O_2_^−^, holes in the valence band (h^+^), and •OH	95% of RhB and 77.6% of TCs by 2%Gd/2%Pt-Bi_2_MoO_6_	[67]
Eu-doped Bi_2_WO_6_ composites	RhB	1 g/L	10 mg/L	60	Visible light, 25 °C, addition of H_2_O_2_	Production of OH radicals	98%	[63]
Ln1/Ln2 co-doped Bi_2_MoO_6_ photocatalysts (Ln1/Ln2 = Tb/Eu, Dy/Sm, Er/Nd)	RhB	1 g/L	12 mg/L	240	UV visible light	Production of OH, holes (h^+^) and superoxide radicals O_2_^−^	95.9% by 3%Tb/3%Eu, 98.5% by 3%Dy/3%Sm and 91.6% by 2%Er/2%Nd	[75]
Gd^3+^ doped Bi_2_MoO_6_ nanoplate crystals	RhB	0.5 g/L	20 mg/L	10	Visible light, 20 °C	Production of OH and holes (h^+^)	84% by 6%Gd/Bi_2_MoO_6_	[68]
Gd^3+^/TiO_2_ and Gd_2_O_3_/TiO_2_ NPs	MV/RhB	0.2 g/L for MV and 0.1 mg/L for RhB	25 mg/L/30 mg/L	60	UV visible light	Production of •O_2_^−^, holes in the valence band (h^+^), and •OH	97.9% by 2.5% Gd^3+^/TiO_2_	[69]
Sm-doped Bi_2_MoO_6_ photocatalyts	RhB	0.2 g/L	5 mg/L	50	Visible light	Production of •O^2−^ and holes (h^+^)	89% by 0.8% Sm-doped Bi_2_MoO_6_	[57]

**Table 5:** REE catalysts used for herbicide, alkaloid, cyanide, organic acid, and phenol degradation.

Catalyst Doped REE	Target	Dose of Catalyst	Dose of Target	Time (min)	Conditions	Proposed Mechanism	Removal (%)	Reference
Ti-LaFeO_3_	Carbofuran	700 mg/L	7 mg/L	180	Adding H_2_O_2_, pH = 3.0	Production of •OH	91%	[76]
Ti-substituted LaFeO_3_	4-Chlorophenol	0.5 g/L	25 mg/L	-	Circumneutral pH, ambient atmospheric pressure and temperature, UV-A light, addition of H_2_O_2_	Production of •OH	100% by 3.2% Ti	[77]
Fe_3_O_4_-CeO_2_	Catechol	1 g/L	10 mg/L	60	Adding H_2_O_2_, room temperature, pH = 2.4	Production of ROS	100% by Fe_3_O_4_-CeO_2_ (15 wt%)	[79]
Ce-Sm, Ce-La, Ce-Zr	Oxalic acid/Aniline/C.I. Reactive Blue 5	-	C0,oxalic acid = C0,aniline = 1 mM, C0,dye = 50 mg L^−1^	180/30/15	pH0,oxalic acid ≈ 3.0, pH0,aniline ≈ 6.5, pH0,dye ≈ 5.5, constant gas (oxygen + ozone) flow rate and constant inlet ozone concentration, 25 °C	Production of •OH	100% of oxalic acid with more than 25% of Sm, La or Zr/100% of aniline by Ce0.75Zr0.25O_2_/100% by Ce0.75Zr0.25O_2_	[80]
Fe_3_O_4_/CeO_2_ composite	4-Chlorophenol	2 g/L	0.78 mM	90	Addition of H_2_O_2_, T = 30 °C, pH = 3.0	Production of •OH	100%	[81]
ZnO nanoparticles doped with Ce	Cyanide	4 g/L	-	60	UV-A light or natural sunlight, pH = 12.5	Production of •OH	-	[84]
Sm/ZNRs	Phenol	1 g/L	20 mg/L	480	Visible light, room temperature	Production of •OH radicals	89.5% by 1 wt% Sm/ZNRs	[85]
Cryptomelane doped with Eu	Ethanol	50 mg	-	-	Dark, T = 175–200 °C	Production of O_2_^−^	100%	[86]
Cerium nanotubes catalysts (CeNTs)	NOx	-	600 mg/L	600	Addition of CH_2_Cl, 200 °C	Production of •OH	100% by 10% Nb-CeNTs	[82]
Gd/Pt-Bi_2_MoO_6_ composites	4-Chlorophenol	1 g/L	15 mg/L	300	UV visible light	Production of •O_2_^−^, holes in the valence band (h^+^), and •OH	80% by 2%Gd/2%Pt-Bi_2_MoO_6_	[49]
Ln1/Ln2 co-doped Bi_2_MoO_6_ photocatalysts (Ln1/Ln2 = Tb/Eu, Dy/Sm, Er/Nd)	Phenol	1 g/L	15 mg/L	300	UV visible light	Production of •O_2_^−^, holes in the valence band (h^+^), and •OH	76.2%, 79.1% and 70.7% by 3%Tb/3%Eu, 3%Dy/3%Sm and 2%Er/2%Nd, respectively	[75]
La-doped TiO_2_ photocatalyst	Acetone/NO	-	1000 ppb/500 ppb	30	UV visible light	Production of •O_2_^−^ and •OH	38% of acetone and 98% of NO by 0.5%La-TiO_2_	[78]

## Data Availability

Not applicable.
